# MicroRNAs—A Promising Tool for Asthma Diagnosis and Severity Assessment: A Systematic Review

**DOI:** 10.3390/jpm12040543

**Published:** 2022-03-29

**Authors:** Mohammed Aref Kyyaly, Elena Vladimirovna Vorobeva, Dilini M. Kothalawala, Wei Chern Gavin Fong, Peijun He, Collin L. Sones, Mohammad Al-Zahrani, Tilman Sanchez-Elsner, Syed Hasan Arshad, Ramesh J. Kurukulaaratchy

**Affiliations:** 1 Clinical and Experimental Sciences, Faculty of Medicine, University of Southampton, Southampton SO16 6YD, UK; aref.kyyaly@solent.ac.uk (M.A.K.); e.v.vorobeva@soton.ac.uk (E.V.V.); gavinfwc@doctors.org.uk (W.C.G.F.); moja1g15@southamptonalumni.ac.uk (M.A.-Z.); t.sanchez-elsner@soton.ac.uk (T.S.-E.); s.h.arshad@soton.ac.uk (S.H.A.); 2 The David Hide Asthma and Allergy Research Centre, Isle of Wight PO30 5TG, UK; 3 Biomedical Science, Faculty of Sport, Health and Social Sciences, Solent University Southampton, Southampton SO14 0YN, UK; 4 NIHR Southampton Biomedical Research Centre, University Hospitals Southampton, Southampton SO16 6YD, UK; d.kothalawala@soton.ac.uk; 5 Human Development and Health, Faculty of Medicine, University of Southampton, Southampton SO16 6YD, UK; 6 Optoelectronics Research Centre, University of Southampton, Southampton SO17 1BJ, UK; p.he@soton.ac.uk (P.H.); cls@orc.soton.ac.uk (C.L.S.); 7 Faculty of Applied Medical Sciences, Al-Baha University, Al Baha 65731, Saudi Arabia

**Keywords:** asthma, asthma severity, biomarker, diagnosis, micro RNA

## Abstract

Micro RNAs (miRNAs) are short, non-coding RNAs (Ribonucleic acids) with regulatory functions that could prove useful as biomarkers for asthma diagnosis and asthma severity-risk stratification. The objective of this systematic review is to identify panels of miRNAs that can be used to support asthma diagnosis and severity-risk assessment. Three databases (Medline, Embase, and SCOPUS) were searched up to 15 September 2020 to identify studies reporting differential expression of specific miRNAs in the tissues of adults and children with asthma. Studies reporting miRNAs associations in animal models that were also studied in humans were included in this review. We identified 75 studies that met our search criteria. Of these, 66 studies reported more than 200 miRNAs that are differentially expressed in asthma patients when compared to non-asthmatic controls. In addition, 16 studies reported 17 miRNAs that are differentially expressed with differences in asthma severity. We were able to construct two panels of miRNAs that are expressed in blood and can serve as core panels to further investigate the practicality and efficiency of using miRNAs as non-invasive biomarkers for asthma diagnosis and severity-risk assessment, respectively.

## 1. Introduction

Asthma is a high morbidity chronic condition affecting over 300 million people worldwide and causing 461,000 deaths globally in 2019 [1]. This global disease burden is expected to rise to 400 million cases by 2025 [2]. It is recognised that asthma is a heterogeneous state composed of numerous clinical phenotypes which may have different underlying biological pathways. Given the absence of gold standard tests to diagnose asthma, most guidelines concur that asthma remains a clinical diagnosis. However, such diagnostic approaches are open to subjective variation. Severe asthma is defined by ATS (American Thoracic Society)/ERS (European Respiratory Society) guidelines as asthma requiring treatment with high-dose inhaled corticosteroids plus a second controller and/or systemic corticosteroids to prevent it from becoming ‘‘uncontrolled’’ or that remains ‘‘uncontrolled’’ despite this therapy [3]. It places a significant burden on both individual patients and wider healthcare resources. There is a clear need to develop diagnostic biomarkers both to aid asthma diagnosis and identify phenotypes with a higher severity risk [4,5].

MicroRNAs (miRNAs) are small non-coding RNAs (Ribonucleic acids), 22–25 nucleotides long, acting through RNA-induced silencing complexes to post-transcriptionally regulate mRNAs (messenger RNA) containing complementary sequences. Highly stable circulating miRNAs occur in biological fluids including peripheral blood and are potential biomarkers [6,7] for diagnosis, prognosis, and disease monitoring [8]. Growing evidence indicates that miRNAs are differentially expressed in asthmatics compared to non-asthmatics and have immunoregulatory effects [9,10,11,12,13,14]. Differences in both exhaled breath condensate (EBC) and bronchoalveolar lavage (BAL) fluid miRNA profiles occur in asthmatics compared to healthy controls. miR-570-3p has shown diverse effects on cytokine expression in human airway epithelial cells while serum and EBC levels correlate inversely with lung function [15].

A set of miRNAs assessed in blood were recently reported and could classify asthmatics into two clusters by blood eosinophil numbers and periostin concentration [16]. Differential serum expression has also been shown between asthma and Chronic Obstructive Pulmonary Disease for multiple miRNAs [17].

Given presence and stability in various tissues, miRNAs are attractive potential biomarkers to aid in the diagnosis of asthma, identification of distinct asthma phenotypes, and stratification of asthma severity-risk.

## 2. Objectives

The aim of this systematic review is to establish which miRNAs have been reported to be differentially expressed in asthma patients and therefore have potential as biomarkers to improve the clinical diagnosis, phenotyping, and severity-risk assessment of asthma.

This systematic review reports the known miRNAs (to date) that show a significant fold difference in expression in different tissues and body fluids including any changes in the expression (up-/downregulation) as well as the presence or absence of those miRNAs to indicate an association with asthma. Studies reporting miRNAs associated with asthma in both adults and children were included. miRNAs in animal models were excluded from this review.

## 3. Methods

This systematic review (PROSPERO registration number: CRD42021226834) was conducted following the guidelines reported in the Preferred Reporting Items for Systematic reviews and Meta-Analyses (PRISMA) statement [18].

### 3.1. Search Strategy

An electronic search of three databases, MEDLINE, EMBASE, and SCOPUS, was performed on 14 September 2020. Free-text and MeSH terms were used to identify articles related to miRNAs differential expression level in asthma (Supplementary Table S1).

All articles underwent a two-stage duplicate removal process first electronically using EndNote X8.2 followed by the manual removal of remaining duplicates. Two independent reviewers conducted a title and abstract screening to assess the relevance of the remaining articles. Discrepancies were resolved through discussion among the reviewers. A full-text and additional screening of citations in selected papers of reported miRNAs differentially expressed in asthma were conducted. Identified studies underwent data extraction.

### 3.2. Eligibility

#### 3.2.1. Inclusion Criteria

Full-text manuscripts published up to 14 September 2020 were reviewed for relevance. Studies were included if recruiting patients with asthma, diagnosed according to one of the following internationally recognised guidelines: BTS (British Thoracic Society), GINA (Global Initiative for Management of Asthma), GEMA (Spanish Asthma Management Guidelines), GDPBAP (Guidelines for Diagnosis and Prevention of Bronchial Asthma in Pediatric Group), or ATS/ERS (American Thoracic Society/European Respiratory Society) or using robust clear diagnostic criteria and reporting at least one miRNA related to asthma diagnosis in humans or reporting at least one miRNA related to asthma severity or phenotype in humans. Novel or updates to previously reported miRNAs/miRNA panels, related to asthma diagnosis/severity, were also included. Both adult and childhood asthma studies were included. Publications reporting miRNAs related to asthma diagnosis/severity in animal models were included only if they were validated in human participants.

#### 3.2.2. Studies Were Excluded If

(1) The study was not in English; (2) the type of the research was a review, case report, or conference summary; (3) the publication was reporting miRNAs related to asthma diagnosis/severity in animal models only; and/or (4) the publication was reporting/comparing miRNAs level between asthma and other respiratory diseases e.g., COPD, allergic rhinitis.

### 3.3. Study Selection

The titles and abstracts of all the records returned by the literature search were independently reviewed by two reviewers for each study (MAK, DMK, WCGF, and EV) to identify potentially relevant studies.

Searches of study bibliographies were conducted to identify additional studies. Using the pre-specified inclusion/exclusion criteria, these two reviewers then independently reviewed the full texts of potentially relevant studies for inclusion in the review.

Disagreements were discussed and resolved by mutual discussion between researchers, but in the event of non-consensus, it was planned that a third reviewer would be involved.

### 3.4. Data Extraction

The extracted data consisted of two parts: the first part contained basic information such as first author, the year and region of publication, the numbers and ages of participants, and the details of differentially expressed miRNA, including the specific miRNA types, the sources of researched samples, detection methods of reported miRNAs, and expression trends between cases and controls. The second part classified the miRNAs according to their specified use as diagnostic biomarkers for asthma/no-asthma, mild/moderate/severe, or allergic/non-allergic asthma.

### 3.5. Quality Assessment and Risk of Bias for Publications

Included papers were assessed for quality and bias by two researchers (MAK and EV) using the Quality Assessment of Diagnostic Accuracy Studies-2 (QUADAS-2) tool through resources available at the QUADAS-2 website https://www.bristol.ac.uk/population-health-sciences/projects/quadas/resources/with (accessed on 14 February 2021) for levels of “high”, “low”, and “unclear” risk of bias.

## 4. Results

### 4.1. Publication Selection

**Figure 1:**
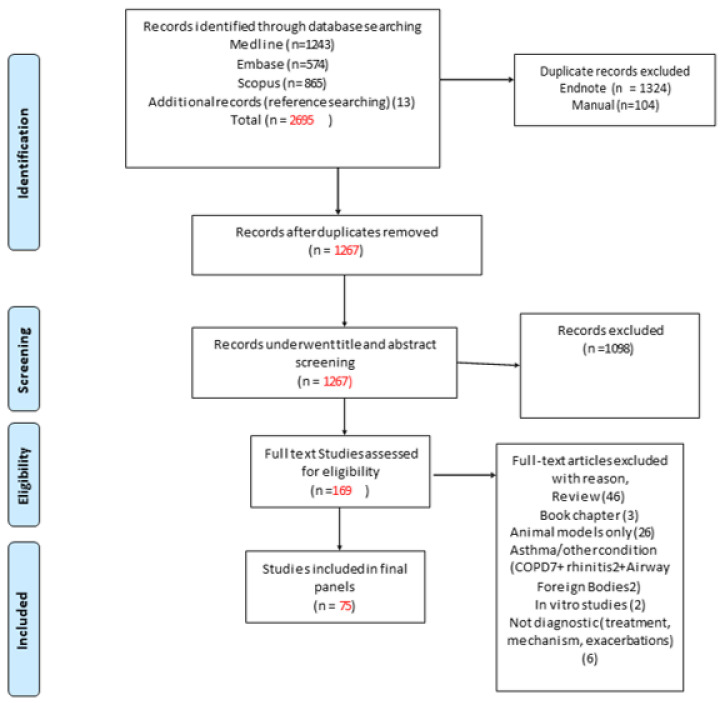
PRISMA flow diagram detailing search results [18].

The initial literature search identified 2695 articles (Figure 1). Following the removal of 1428 duplicate articles, 1267 articles underwent title and abstract screening. That screening process identified 169 articles for full-text review. Of those, 75 studies were deemed relevant while 94 studies were excluded (11 reporting other conditions such as rhinitis/COPD, 26 studies were on animal models only, 49 reviews and book chapters, 6 studies were not diagnostic, and 2 were in vitro studies). The final 75 studies were classified into two categories based on the clinical use of miRNAs for either asthma diagnosis or asthma phenotyping. For each category, the miRNAs were grouped according to the source of sample to exhaled breath condensate (EBC), Bronchoalveolar lavage (BAL), bronchial epithelial cells (BEC), lung tissue, and blood (serum, plasma, lymphocytes, PBMCs (peripheral blood mononuclear leukocytes), or whole blood). All selected studies were prospective.

### 4.2. Quality Assessment (Risk of Bias)

**Figure 2:**
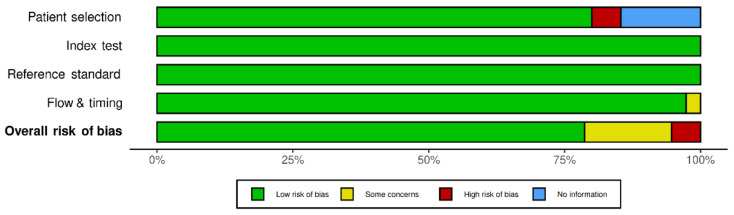
Quality assessment (Methodological quality summary graph.) of the 75 included studies using the tool of Quality Assessment of Diagnostic Accuracy Studies-2 (QUADAS-2).

The final 75 studies selected were assessed for risk of bias using QUADAS-2 quality evaluation. Figure 2 shows the details of the assessment. The results showed that there were four studies with a high risk of bias. The main source of risk of bias in these studies was from patient selection, as we considered using physician diagnoses or self-reporting without specifically defined robust criteria for diagnosis to be a potential source of risk. However, these studies were included in this systematic review, given that an asthma diagnosis of some form had been applied.

Of these 75 studies, 9 studies reported miRNAs differential expression among different severity groups (severity-risk assessment only), and 57 studies reported the differential expression of miRNAs between asthma patients and non-asthmatic subjects (diagnostic only). Nine studies reported miRNA’s differential expression among different severity groups and between non-asthma subjects and asthma patients (both diagnostic and severity-risk assessment).

To study the miRNAs reported in the selected 75 publications, we created two categories for miRNAs:

- miRNAs for asthma diagnosis.
- miRNAs for severity-risk assessment.

### 4.3. miRNAs for Asthma Diagnosis

**Figure 3:**
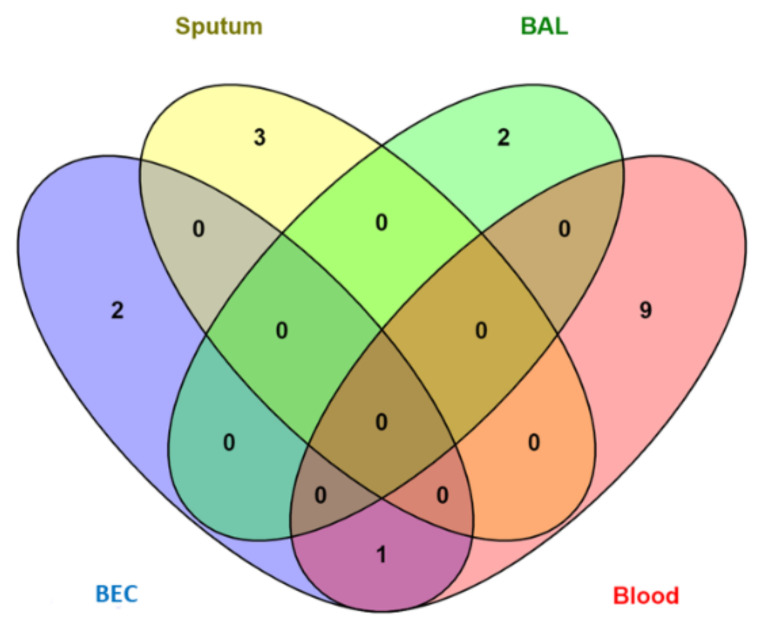
Distribution of diagnostic miRNAs for asthma among different tissues (BAL, BEC, Sputum, EBC, and blood).

From the included studies, 66 studies reported a difference in miRNAs expression between asthmatic patients and non-asthmatic control groups. miRNAs were measured in five tissues and body fluids: blood (serum, plasma, or PBMCs), sputum, BAL, BEC, or EBC. The results showed that 164, 41, 30, 19, and 5 miRNAs were reported to have significantly different expression levels in the blood (and its components), BECs, BAL, sputum, and EBC, respectively. Figure 3 shows the numbers of the differentially expressed miRNAs in asthma patients compared to non-asthmatic controls found in various tissues and body fluids.

Eight miRNAs (miR-126, let-7e, miR-224, miR-143, miR-181b-5p, miR-378, miR-27b-3p, and miR-221) were shared between blood and BEC. Six miRNAs (miR-223-3p, miR-142-3p, miR-125b, miR-16-5p, miR-4284, and miR-199a-5p) were shared between blood and sputum. Four miRNAs (let-7b, miR-339-3p, miR-615-3p, and miR-30a) were shared between blood and BAL. Two miRNAs (miR-126-3p and miR-21-5p) were shared between blood and EBC. Two miRNAs (miR-19a and miR-27a) were shared between BEC and BAL. Two miRNAs (let-7a and miR-21) were shared among BEC, BAL and blood. One miRNA (miR-221-3p) was shared among BEC, sputum, and blood. Finally, only one miRNA (miR-155) was shared among blood, BAL, airway cells, and sputum.

We conducted a qualitative analysis from all included studies. Firstly, for diagnostic miRNAs, we found 223 miRNAs reported as differentially expressed between asthmatic patients and controls in all included publications. Then we created a category for miRNAs that were reported in more than two articles. This resulted in 10 miRNAs (miR- 126, miR-155, let-7 family, miR-21, miR-125b, miR-146a, miR-192, miR-15a, miR-30a, and miR-98) (Table 1).

The results also showed excellent consistency within specific tissues in terms of up- or downregulation of the studied miRNAs. The only inconsistency was in miR-21 from BAL17,18 where this miRNA was upregulated in BAL of asthmatic patients from one study18 and downregulated in BAL samples of asthma patients when compared to controls from another study [29]. This discrepancy can be explained because total small RNAs were extracted in the first study [28], while only exosomes from BAL were used in the other study [29]. Otherwise, this miRNA was upregulated in all other studied tissues (plasma, serum, and BEC) [23,24,30,31,32,33]. Of these 10 miRNAs, miR-155 was upregulated in serum24-26 and downregulated in airways (BAL, sputum, and nasal biopsies) [22,27,28,47,48]. Let-7 family (a-i) and miR-30a were downregulated in all studied tissues (BAL, serum, BEC, and lymphocytes) [9,21,28,29,44,48]. miR-192 and miR-15a were only studied in the blood (plasma, serum, lymphocytes, and whole blood) and also downregulated in asthma patients [21,40,41,42,43]. In addition, miR-126 was upregulated in plasma, serum, and BEC [19,20,23,34,49] and downregulated in lymphocytes and nasal biopsies [21,22]. miR-125b was upregulated in plasma and serum [20,41] and downregulated in sputum and lymphocytes [21,35]. miR-146a was upregulated in plasma and serum [25,32,36,37] and downregulated in PBMCs and EBC [38,39]. Finally, miR-98, was only studied in blood, and it was upregulated in B lymphocytes and whole blood [45,46] while downregulated when measured in total lymphocytes [21].

From these results, we conclude that miRNA expression patterns are consistently expressed in the blood of patients with asthma, enabling us to propose a panel of up/down-regulated miRNAs that can potentially be used to confirm/support asthma diagnosis (Table 2).

### 4.4. miRNAs for Asthma Severity-Risk Assessment

**Figure 4:**
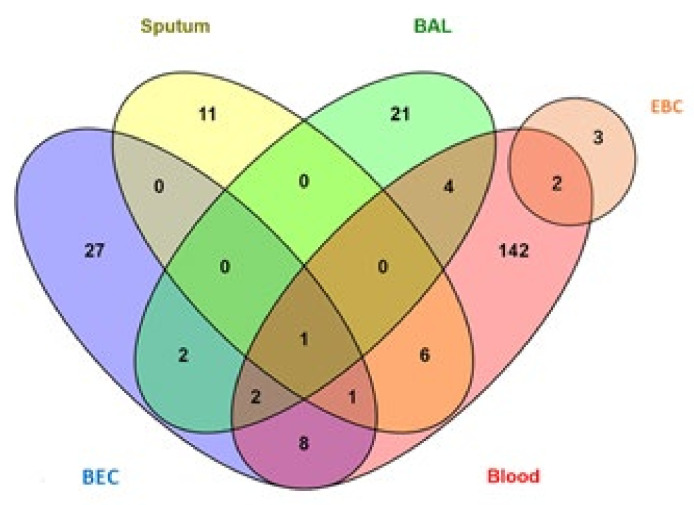
Distribution of miRNAs for asthma severity-risk among four tissues (BAL, BEC, sputum, and blood).

Only 16 papers of the 75 included studies studied miRNAs differential expression among different asthma severity groups. Four of these studies [32,42,46,50] either reported no difference or non-significant difference in miRNAs expression among different severity groups. The remaining 12 studies reported a significant difference in the expression of 17 miRNAs from different tissues that can help in the severity assessment of asthma patients (Table 3). Only one miRNA (Let-7a) was studied in two different tissues (blood/plasma and BEC) and was downregulated in both tissues of severe asthmatic patients compared to mild asthma patients (inversely related to asthma severity). Figure 4 shows the numbers of miRNAs from different tissues and body fluids found to have significantly different expressions for the severity of asthma.

Similar to the diagnostic panel we suggested above (Table 2) and guided by the data obtained from the studied publications, we can here suggest a panel of six miRNAs in the blood that can serve as a non-invasive test to assess asthma severity-risk. Table 4 lists these miRNAs.

## 5. Discussion

**Figure 5:**
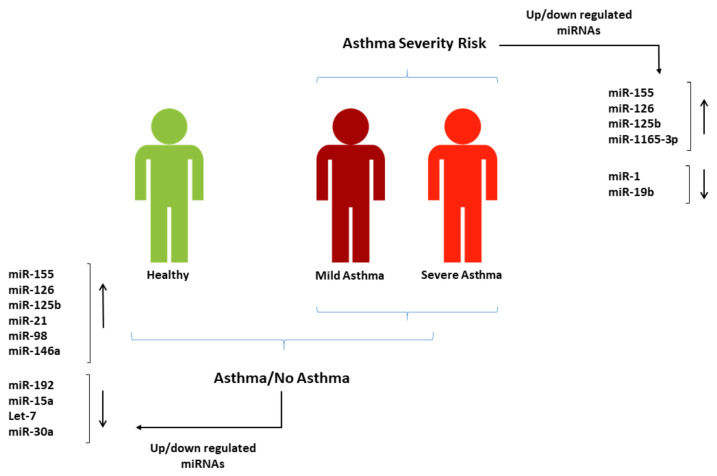
Suggested miRNA panels from the studies included in this systematic review that can be used in blood for asthma diagnosis and severity-risk assessment.

In this study, we conducted a qualitative systematic review to evaluate the diagnostic and severity-risk assessment value of miRNAs in asthma. We adopted a novel approach where we evaluated the diagnostic and severity-risk assessment value of miRNAs in asthma to propose two corresponding panels of miRNAs of potential clinical utility in asthma for the first time. We found more than 200 miRNAs from all included studies that were differentially expressed in asthma. Though the expression levels and trends varied in different publications, we constructed a panel of 10 diagnostic miRNAs, each reported in more than two studies with significant differential expressions between those with and without an asthma diagnosis. These miRNAs could be potentially used to develop a non-invasive test to support asthma diagnosis, as their expression differed significantly between people with asthma and those without asthma. Similarly, 17 miRNAs from the included studies were differentially expressed in the studied tissues with respect to asthma severity, and we constructed another panel of 4 differentially expressed miRNAs in the blood that can potentially help assess asthma severity-risk. Figure 5 summarises the two miRNA panels. 

There were potential limitations to this study. In that context, there were a relatively small number of studies reporting miRNAs specifically related to asthma. Furthermore, there remains limited knowledge about the functional roles of many miRNAs in the pathogenesis and clinical expression of asthma. In that regard, limited phenotyping information was available in most studies to draw such conclusions. In addition, some information such as therapeutic status, time points, or intervention were not mentioned in many studies, so we could not include these elements in our selection criteria. However, there were also cores strengths to our approach. We followed PRISMA guidelines to report studies and The Quality Assessment of Diagnostic Accuracy Studies-2 (QUADAS-2) to evaluate the risk of bias. This tool is used to study the applicability of diagnostic accuracy studies in systematic reviews. It also provides an important foundation for researchers to design and assess high-quality studies.

The functional relevance of some miRNAs identified in this systematic review remains unclear, whilst other identified miRNAs have already been shown to have associations with specific aspects of asthma pathogenesis or clinical expression. Of the 10 miRNAs in the final “diagnostic panel”, 6 miRNAs (miR-21, miR-125b, miR-146a, miR-30a, miR-155, miR-126) have already characterised functional effects of relevance to asthma. Of the four miRNAs in the final “severity-risk” assessment panel, three have existing characterised functional effects of relevance to asthma.

In this context, miR-146a was found to be of diagnostic value for asthma as it was upregulated in the blood of asthmatics and associated with markers of poor asthma control, such as the Asthma Control Questionnaire score [36]. Expression of this miRNA was also found to inhibit the proliferation and promote the apoptosis of Bronchial Smooth Muscle Cells, providing a novel clinical marker for the diagnosis and stratification of asthma [37].

A role of miR-30a in suppressing airway fibrosis and autophagy by targeting ATG5 (autophagy-related gene 5) has been shown [44]. Thus, miR-30a expression inversely correlates with autophagy in asthma, supporting the hypothesis that lower miR-30a levels lead to the overexpression of ATG5, which promotes asthma progression. Conversely, increased miR-21 expression has been previously associated with asthma development, mainly due to its targeting of IL-12p35 highlighting the multi-functional roles of miRNAs in several cell types contributing synergistically to asthma pathology [58]. Functionally, miRNA-21 can stimulate a Th2 response by two different mechanisms [24]. The first mechanism is mediated by the ability of miRNA-21 to inhibit IL-12 gene expression, resulting in the inhibition of Th1 functions, including the decreased release of IFN-γ. Low IFN-γ levels lead to unrestricted Th2 activation and elevated Th2 cytokines. Th2 cytokines can additionally augment miRNA-21-mediated responses [59]. Second, miRNA-21 can directly induce differentiation of T cells towards Th2 lineage by escalating GATA-3 and IL-4 expression immediately after T-cell activation [23,60,61].

Three miRNAs miR-155, miR-126, and miR-125b were shared between the two panels and will be discussed further. These miRNAs (together with other miRNAs also reported in our diagnostic panel) are thought to be influential in miR-mediated Toll-like receptor (TLR) signalling and cytokine signalling in asthma [62].

miR-155 is one of the most studied miRNAs. It is implicated in a variety of diseases and is widely studied in asthma [25]. miR-155 is a critical regulator of type 2 innate lymphoid cells in murine models of allergic airway inflammation [63] and was shown to be differentially expressed in the airways of allergic asthmatic individuals compared to healthy controls [27]. It was also found that the increased expression of miR-155 enhances mucus secretion and regulates the secretion of IL-4, IL-5, IL-13, and IL-17a [64,65].

A recent study found a highly significant correlation between the increased expression of miR-125b and CRP/IgE levels (r = 0.86/r = 0.68; *p* < 0.0001) in asthma patients, indicating the potential relevance of this miRNA for particular asthma phenotypes.

It has been reported [19] that the overexpression of miR-126 in bronchial epithelial cells can promote increased levels of the Th2 cytokine IL-13, suggesting that miR-126 should be studied as a factor related to the excessive activation of Th2 cells in asthmatic children. It has been also found that the relative level of miR-126 was independently associated with the proportion of Th17 cells, indicating that the mechanism of miR-126 overexpression in promoting asthma may also be related to the proportion of Th17 cells. The same study [19] also showed that the relative level of miR-126 in the peripheral blood of asthma children was associated with the degree of asthma severity, emphasising its potential role as a severity risk marker.

One noteworthy finding was that some miRNAs can be upregulated in one tissue compartment but downregulated in another compartment. For example, miR-155 is upregulated in serum and downregulated everywhere else (BAL, sputum, and nasal biopsies) [22,27,28,47]. This differential downregulation suggests that some miRNAs can be used as a biomarker but that we cannot yet necessarily draw functional conclusions about their role in asthma pathophysiology or clinical expression.

## 6. Conclusions

Despite the increasing evidence that miRNAs play important roles in asthma, the studies performed so far were mostly conducted in in vitro or animal models of asthma, and evidence in humans remains limited. Thus, adequately powered studies are now needed to improve our insight into the role of miRNAs in relevant human cells and tissues [66]. Here, we reviewed human studies available so far and selected panels of miRNAs that can serve as core panels to investigate the practicality and efficiency of using microRNAs as non-invasive biomarkers in blood for asthma diagnosis and severity-risk assessment. Further studies to assess the clinical utility of such diagnostic and severity-risk miRNA panels in asthma as well as functional roles of those miRNAs in the pathogenesis and clinical expression of asthma should be a future research focus.

## Figures and Tables

**Table 1:** miRNAs studied in more than two publications to be of diagnostic value.

miRNA	Tissue	Up-/DownRegulated	Ref
**miR-126**	Plasma	+	[19,20]
	Serum	+	[20]
	Lymphocytes	-	[21]
	Nasal biopsy	-	[22]
	EBC	+	[23]
**miR-155**	Serum	+	[24,25,26]
	Sputum	-	[27]
	Nasal biopsy	-	[22]
	EBC	-	[27]
	BAL	-	[28]
**Let-7 family**	BAL	-	[29,28]
	Serum	-	[9]
	EBC	-	[27]
	Lymphocytes	-	[21]
**miR-21**	BAL	-/+	exosomes [17]/total small RNA [18]
	Plasma	+	[30,31,32]
	Serum	+	[33,24]
	EBC	+	[23]
**miR-125b**	Plasma	+	[20]
	Serum	+	[34]
	Sputum	-	[35]
	Lymphocytes	-	[21]
**miR-146a**	Serum	+	[25,36]
	Plasma	+	[32,37]
	PBMCs	-	[38]
	EBC	-	[39]
**miR-192**	Blood	-	[40]
	Plasma	-	[41]
	Lymphocytes and CD4+	-	[21,41]
**miR-15a**	Serum	-	[42]
	Lymphocytes and CD4+	-	[21,43]
**miR-30a**	Serum	-	[44]
	BAL	-	[28]
	Lymphocytes	-	[21]
**miR-98**	B cells	+	[45]
	Blood	+	[46]
	Lymphocytes	-	[21]

PBMC: Peripheral blood mononuclear cells. BAL: bronchoalveolar lavage. BEC: bronchial epithelial cells. EBC: exhaled breath condensate. +: upregulated. -: downregulated.

**Table 2:** miRNAs studied in the blood that can serve as a diagnostic panel.

miRNA	+/-	Sample	Ref
miR-126	+	Plasma and Serum	[19,20,49]
miR-155	+	Serum	[24,25,26]
miR-21	+	Plasma and Serum	[24,30,31,32,33]
miR-125b	+	Plasma and Serum	[20,34]
miR-98	+	Blood	[46]
miR-146a	+	Plasma and Serum	[25,32,36,37]
miR-192	-	Plasma	[41]
miR-15a	-	Serum	[42]
Let-7	-	Serum	[9]
miR-30a	-	Serum	[44]

+: upregulated. -: downregulated.

**Table 3:** miRNAs studied in publications to have altered levels due to asthma severity.

miRNA	+/-	Sample	Ref
miR-320a	+	Eosinophils	[16]
miR-185-5p	+	Eosinophils	[16]
miR-144-5p	+	Eosinophils	[16]
miR-629-3p	+	Sputum (Supernatant)	[51]
miR-223-3p	+	Sputum (Supernatant)	[51]
miR142-3p	+	Sputum (Supernatant)	[51]
miR-126	+	Plasma	[19]
miR-155	+	Plasma	[52]
miR-125b	+	Serum (exosomes)	[34]
miR-19a	+	BEC	[53]
miR-1165-3p	+	Serum	[54]
miR-200b	-	BAL	[28]
miR-1	-	Plasma	[55]
miR-19b	-	Serum	[56]
Let-7a	-	BEC/plasma	[48,52]
miR-744	-	BEC	[57]
miR-200c	-	BAL	[28]

BAL: bronchoalveolar lavage. BEC: bronchial epithelial cells. +: upregulated. -: downregulated.

**Table 4:** miRNAs studied in the blood that can serve as severity-risk assessment panel.

miRNA	+/-	Sample	Ref
miR-126	+	Plasma	[19]
miR-155	+	Plasma	[52]
miR-125b	+	Serum (exosomes)	[34]
miR-1165-3p	+	Serum	[54]
miR-1	-	Plasma	[55]
miR-19b	-	Serum	[56]

+: upregulated. -: downregulated.

## Data Availability

Not applicable.

## Supplementary Materials

The following supporting information can be downloaded at https://www.mdpi.com/article/10.3390/jpm12040543/s1, Table S1: Search criteria and the results for MEDLINE, Embase and SCOPUS databases.
