# Supplementary material for: MicroRNAs—A Promising Tool for Asthma Diagnosis and Severity Assessment: A Systematic Review
Source: J Pers Med. 2022 Mar 29;12(4):543. doi: 10.3390/jpm12040543 (PMC9030707; doi:10.3390/jpm12040543)
Supplement: Supplementary file 1 [file jpm-12-00543-s001.zip › jpm-1615987-supplementary.pdf]

Table S1. Search criteria and the results for MEDLINE, Embase and SCOPUS databases.

A- MEDLINE (Ovid): 1992-now (14/09/2020)

Step	Term	No. of articles	Comments
1	Asthma/	125743	Map Term to Subject Heading ticked
2	Asthma*	180907	
3	1 or 2	180904	
	microRNA*/		
4	miRNA*	68259	
5	Micro RNA*	3267	
6	microRNA*	107926	
7	Small RNA*	13424	
8	4 or 5 or 6 or 7	124021	
9	3 and 8	560	

B- Embase (Ovid): 1992-now (14/09/2020)

Step	Term	No. of articles	Comments
1	Asthma*	273980	Map Term to Subject Heading ticked
2	Asthma*	313480	
3	1 or 2	313480	
4	miRNA*	93797	
5	Micro RNA*	6334	
6	microRNA*	168658	
7	Small RNA*	16272	
8	4 or 5 or 6 or 7	179891	
9	3 and 8	1238	

C- SCOPUS: 1992-now (14/09/2020)

Step	Term	No. of articles	Comments
1	Asthma*	204290	Document search Ti, Ab, Key
2	miRNA*	73539	Document search Ti, Ab, Key
3	microRNA*	135964	Document search Ti, Ab, Key
4	“micro RNA*”	4262	Document search Ti, Ab, Key
5	“small RNA*”	14574	Document search Ti, Ab, Key
6	2 or 3 or 4 or 5	149187	Document search Ti, Ab, Key
7	1 and 6	860	Document search Ti, Ab, Key
